# Bayesian spatial analysis of a national urinary schistosomiasis questionnaire to assist geographic targeting of schistosomiasis control in Tanzania, East Africa

**DOI:** 10.1016/j.ijpara.2007.08.001

**Published:** 2008-03

**Authors:** A.C.A. Clements, S. Brooker, U. Nyandindi, A. Fenwick, L. Blair

**Affiliations:** a Division of Epidemiology and Social Medicine, School of Population Health, University of Queensland, Herston Road, Herston, Qld. 4006, Australia; b Department of Infectious and Tropical Diseases, London School of Hygiene and Tropical Medicine, London, UK; c National Schistosomiasis and Soil-Transmitted Helminth Control Programme, Ministry of Health, Dar es Salaam, Tanzania; d Schistosomiasis Control Initiative, Department of Infectious Disease Epidemiology, Imperial College London, London, UK

**Keywords:** Schistosomiasis, *Schistosoma haematobium*, Haematuria, Questionnaire, Spatial analysis, Bayesian modelling, CAR model, Disease control, Tanzania

## Abstract

Spatial modelling was applied to self-reported schistosomiasis data from over 2.5 million school students from 12,399 schools in all regions of mainland Tanzania. The aims were to derive statistically robust prevalence estimates in small geographical units (wards), to identify spatial clusters of high and low prevalence and to quantify uncertainty surrounding prevalence estimates. The objective was to permit informed decision-making for targeting of resources by the Tanzanian national schistosomiasis control programme. Bayesian logistic regression models were constructed to investigate the risk of schistosomiasis in each ward, based on the prevalence of self-reported schistosomiasis and blood in urine. Models contained covariates representing climatic and demographic effects and random effects for spatial clustering. Degree of urbanisation, median elevation of the ward and median normalised difference vegetation index (NDVI) were significantly and negatively associated with schistosomiasis prevalence. Most regions contained wards that had >95% certainty of schistosomiasis prevalence being >10%, the selected threshold for bi-annual mass chemotherapy of school-age children. Wards with >95% certainty of schistosomiasis prevalence being >30%, the selected threshold for annual mass chemotherapy of school-age children, were clustered in north-western, south-western and south-eastern regions. Large sample sizes in most wards meant raw prevalence estimates were robust. However, when uncertainties were investigated, intervention status was equivocal in 6.7–13.0% of wards depending on the criterion used. The resulting maps are being used to plan the distribution of praziquantel to participating districts; they will be applied to prioritising control in those wards where prevalence was unequivocally above thresholds for intervention and might direct decision-makers to obtain more information in wards where intervention status was uncertain.

## 1 Introduction

Recent estimates have put the number of people at risk of schistosomiasis at 779 million and the number actually infected at 207 million, the vast majority of whom are located in sub-Saharan Africa (SSA) (Steinmann et al., 2006). To help tackle schistosomiasis, new partnerships and global alliances have been formed, with a focus on preventative chemotherapy using praziquantel (World Health Organisation (WHO), 2006). In SSA, schistosomiasis has two forms: urinary schistosomiasis, caused by infection with *Schistosoma haemtatobium*, and intestinal schistosomiasis, caused by *Schistosoma mansoni*. Both forms have a focal distribution in endemic countries, which has been partly explained by a range of climatic, ecological and sociological factors (Brooker, 2007). Such focality means that mass treatment with praziquantel needs to be targeted specifically at high-prevalence communities (Lengeler et al., 2002). Efficient, geographically targeted allocation of limited resources can be facilitated by, and represents the ultimate application of, spatial analysis in the context of parasitic disease control (Hay and Snow, 2006; Brooker and Utzinger, 2007).

Originally developed, administered and tested in Tanzania (Lengeler et al., 1991), questionnaires for self-reported urinary schistosomiasis have been adopted as a rapidly administered, low-cost alternative to parasitological screening of *S. haematobium* infection. They have been validated in a wide range of African settings (Ansell et al., 1997; Booth et al., 1998; Guyatt et al., 1999; Lengeler et al., 2000; Mafe et al., 2000), and are recommended by the WHO for identifying high-prevalence communities in need of mass treatment with anti-schistosomal drugs (Chitsulo et al., 1995). In 2004, the Tanzanian National Schistosomiasis and Soil-Transmitted Helminth Control Programme (NSSCP) conducted the first-ever national schistosomiasis questionnaire survey as part of its planning activities, collecting data from over two and a half million school children in all 21 mainland regions. This provided a unique opportunity to investigate spatial analysis as a tool for resource planning in a national control programme, using the largest schistosomiasis questionnaire study, and perhaps one of the largest tropical disease surveys, ever conducted.

In the current study, a ward-level Bayesian spatial analysis of the questionnaire data was conducted for self-reported schistosomiasis and self-reported blood in urine (BIU), a sensitive and specific clinical sign of urinary schistosomiasis, with the following aims: (i) to derive estimates of prevalence of schistosomiasis in Tanzanian wards that are robust to the influence of varying sample sizes; (ii) to identify clusters of wards with high and low prevalence of urinary schistosomiasis in order to facilitate future epidemiological investigations and geographic targeting of control programmes; and (iii) to quantify the uncertainty surrounding prevalence estimates in order to give decision-makers a more thorough understanding of risks associated with different resource allocation strategies, and to direct future data collection.

## 2 Materials and methods

### 2.1 Control programme

The NSSCP was established in 2003 with support from the Schistosomiasis Control Initiative (SCI, www.schisto.org). Details of the programme are provided in Kabatereine et al. (2006). The programme has responsibility for delivering mass treatment with praziquantel to high-prevalence areas in all 21 mainland regions. It classifies communities on the basis of prevalence of schistosomiasis in school-age children, according to three strategies: (i) in schools where prevalence is <10%, school-age children are treated once upon entering and once upon exiting primary school; (ii) in schools where the prevalence is 10–50%, mass treatment of all school-age children is conducted every other year; and (iii) in schools where the prevalence is >50%, mass treatment of all school-age children is conducted annually. The schistosomiasis morbidity questionnaire has been found to consistently underestimate the true prevalence of infection by approximately 20% (Ansell et al., 1997), leading to a recommendation that a prevalence of self-reported schistosomiasis of 30% be used to define communities where the true prevalence is expected to be above 50% and the NSSCP has adopted this recommendation. As soil-transmitted helminth infections are widespread in Tanzania, albendazole is co-administered with praziquantel. Treatment of school-age children commenced in 2005, in six north-western and five coastal regions.

### 2.2 Questionnaire data

The questionnaire survey is briefly described in Kabatereine et al. (2006) and more detail will be provided in a forthcoming paper, but here we summarize the main features. The survey was administered through the existing infrastructure of the Tanzanian National School Health Programme. District school health coordinators (DSHC) from the education and health departments were educated in how to train teachers to administer the questionnaire (Supplementary File 1) to students in primary school grades one, three and five. Questionnaires were distributed by DSHC to head teachers who, in turn, distributed questionnaires to teachers who administered the questionnaires. All students in eligible grades were asked whether they had experienced schistosomiasis (“kichocho” in Kiswahili) or BIU (“damu katika mkojo” in Kiswahili) in the previous 2 weeks. As with other schistosomiasis morbidity questionnaires, these questions were placed amongst several (in our case, 14) masking questions (e.g. “Did you have malaria in the last 2 weeks?”) to obscure the purpose of the survey and minimise bias. Responses (“yes”, “no” or “don’t know”) were entered into the questionnaire forms. Completed questionnaires were returned to the DSHC and they were subsequently passed back to the coordinator of the NSSCP. Responses were entered into a Microsoft Access database.

School-level prevalence of self-reported schistosomiasis and BIU were compared with parasitological data from 120 schools in four regions of north-western Tanzania (Clements et al., 2006a) and 27 schools in one region of coastal Tanzania (Tanga), and microhaematuria data in 58 schools in four other coastal regions. Prevalence based on parasitological or microhaematuria data was dichotomised according to the intervention thresholds. Area under the curve (AUC) of the receiver operating characteristic (ROC), a plot of sensitivity versus one minus specificity, was used as the test statistic for comparing the questionnaire prevalence to parasitological or microhaematuria prevalence and values of AUC > 0.7 were considered to indicate acceptable discriminatory performance.

The ward was the lowest administrative level for which a digitised map was available (obtained from the International Livestock Research Institute, Nairobi, Kenya) and it was also selected as the administrative unit of intervention by the coordinators of the NSSCP. The data were aggregated at the ward level, with the number of positive and total number of students interviewed (excluding “don’t knows”) calculated for each ward, for both of the questions of interest. The digitised ward map included 2542 wards in Tanzania (excluding Zanzibar), of which 2332 (91.7%) had questionnaire data that could be linked on the basis of the ward name and district.

### 2.3 Spatial analysis

The association of schistosome infection patterns with known ecological factors and the existence of spatial heterogeneity necessitate the accurate accommodation of covariate information and the spatial correlation structure of the data into any statistical analysis. Failure to do the latter results in violation of the important statistical assumption of independence. Additionally, representation of uncertainty in the data and analytical outputs can assist decision-makers to gain an appreciation of the risks associated with data and model-directed decisions for disease control. Bayesian models, which represent the current leading edge in spatial statistics, have become increasingly popular due to their ability not only to incorporate spatial dependence and covariates, but also to fully represent uncertainty in model outputs (Best et al., 2005). It is only recently that the full potential of Bayesian spatial analysis has started to be explored as a resource allocation tool for large-scale schistosomiasis control (Raso et al., 2005, 2006; Yang et al., 2005; Clements et al., 2006a,b).

Ward urbanisation category (rural, urban or mixed rural/urban) and population were available from the 2002 Tanzanian census (www.tanzania.go.tz/census/). Additionally, the following ecological variables were available in 1 km squared raster GIS format: long-term average normalised difference vegetation index (NDVI, a surrogate for rainfall), long-term average land surface temperature (LST), amplitude of NDVI, amplitude of LST and elevation. For a detailed description of how these ecological variables were derived see Hay et al. (2006). The ecological variables were imported into the geographical information system (GIS) ArcView version 9 (ESRI, ESRI, Redlands, CA) and the median value for each ward was calculated.

Initially, a non-spatial, frequentist logistic regression analysis was conducted in Stata version 9 (Stata Corporation, College Station, Texas) to select candidate variables for the Bayesian spatial models. Two variable selection methods were considered: the first where Wald’s *P*-value of ⩽0.20 was the inclusion criterion (the “full” model) and the second where Wald’s *P*-value ⩽0.05 (the “reduced” model) was the inclusion criterion. Non-linear associations between covariates and outcome variables were modelled using quadratic terms and no interactions were considered. Spatially explicit logistic regression models were then constructed in WinBUGS version 14 (MRC Biostatistics Unit, Cambridge, UK). They were of the form

$$\begin{array}{cc} & Y_{i}∼\mathrm{Binomial}(n_{i}\text{,}p_{i})\\ & \mathrm{logit}(p_{i})=\alpha +\overset{p}{\underset{j=1}{\sum }}\beta_{j}\times x_{i\text{,}j}+u_{i}+v_{i} \end{array}$$

where *Y*_*i*_ is the number of positive responses in ward *i*, *n*_*i*_ is the number questioned in ward *i*, *p*_*i*_ is prevalence of positive responses in ward *i*, *α* is the intercept, $\sum_{j=1}^{p}\beta_{j}\times x_{i\text{,}j}$ is a vector of *p* selected independent variables measured in each ward *i* multiplied by their coefficient *β*_*j*_, *u*_*i*_ is a spatial random effect (SRE) and *v*_*i*_ is a non-spatial random effect (NSRE). Non-informative priors were specified for the intercept (uniform prior with bounds −∞, ∞) and the coefficients (normal prior with mean = 0 and precision, the inverse of variance = 1 × 10^−6^). The SRE was modelled using a conditional autoregressive prior structure (Besag et al., 1991), where a simple adjacency matrix was specified with a weight of one given to pairs of wards that had a common border and a weight of zero given to pairs of wards that did not share a border. Both the SRE and NSRE had non-informative priors imposed on their variance (uniform distributions with delimiting values = 1 × 10^−6^ and 1000).

Three simultaneous runs of each model were set up, where the software used Gibbs sampling to sample from the posterior distributions of each variable. A burn-in of 1000 iterations was allowed, followed by 10,000 iterations where values for monitored variables were stored. Diagnostic tests for convergence of the monitored variables were undertaken, including visual examination of history and density plots of the three runs and visual analysis of the Brooks, Gelman and Rubin statistic (Brooks and Gelman, 1998). Convergence was successfully achieved after 10,000 iterations for all variables in both models. The runs were also examined for autocorrelation by visual assessment of the in-built autocorrelation function of WinBUGS. As autocorrelation was apparent for all variables it was decided to thin subsequent sampling by storing every 10th iteration. Sufficient iterations were then run to give a total of 10,000 values stored from the posterior distribution of each variable.

Further model reduction was conducted for the reduced model to exclude variables that were not significantly associated with the outcome variables once spatial correlation was accounted for (LST was removed at this stage). The final full models for both outcome variables included elevation, LST, NDVI and population density with quadratic terms, urbanisation category, amplitude of LST and amplitude of NDVI, and the final reduced models for both outcomes included urbanisation category, elevation and NDVI. The full and reduced models were compared using the deviance information criterion (DIC), where a lower value indicates a better compromise between model fit and parsimony.

## 3 Results

### 3.1 Raw data

In total, data were obtained from 2,586,140 schoolchildren, with an average age of 10.2 years (median 10 years, 5th percentile 7 years, 95th percentile 14 years), in 12,399 schools located in 2373 wards in 116 districts. Overall, the prevalence of self-reported schistosomiasis was 24.8% (23.4% if “don’t know” was treated as “no”) and prevalence of self-reported BIU was 20.5% (19.5% if “don’t know” was treated as “no”). The discriminatory performance of self-reported schistosomiasis and BIU was acceptable where the true prevalence was dichotomised at the 10% intervention threshold in north-western Tanzania (AUC 0.86 and 0.82) and Tanga (AUC 0.91 and 0.89) but not in the other coastal regions (AUC 0.66 and 0.68). The discriminatory performance of both responses was acceptable where the true prevalence was dichotomised at the 50% intervention threshold in north-western Tanzania (AUC 0.83 and 0.81) and the four coastal regions where microhaematuria data were collected (AUC 0.78 and 0.84). This was not assessed in Tanga as all schools had a true prevalence <50%.

**Fig. 1:**
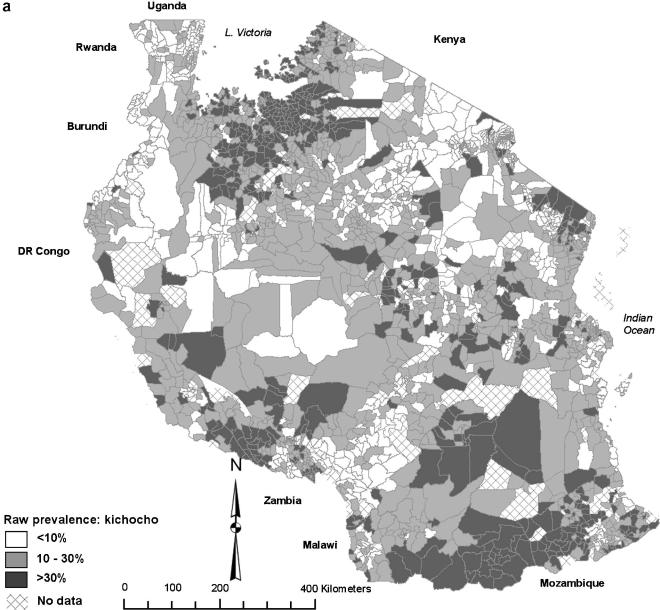

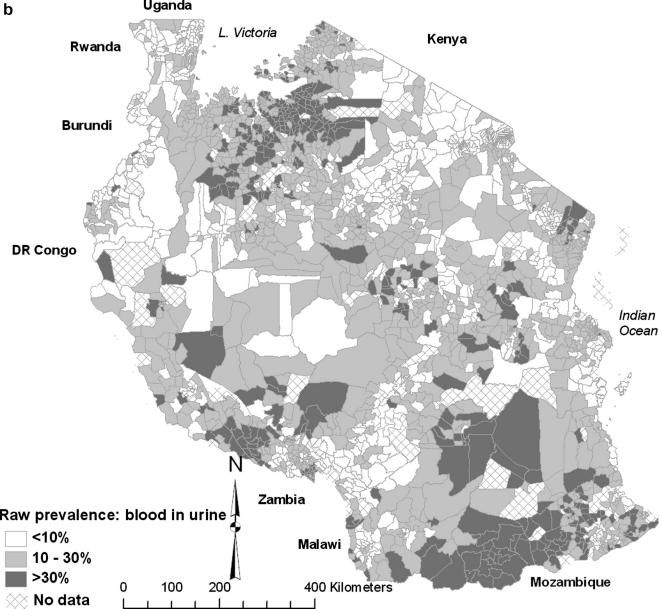
Raw prevalence of self-reported schistosomiasis (a) and blood in urine (b) in Tanzanian wards.

Mapping of the raw data showed that the spatial patterns of ward-level prevalence of self-reported schistosomiasis and self-reported BIU were very similar (Fig. 1). Surprisingly, much of coastal Tanzania, which had previously been identified as a high-prevalence area (Brooker et al., 2001), was found to have a low prevalence of self-reported schistosomiasis and BIU. The prevalence of self-reported schistosomiasis was higher along the eastern coastline of Lake Victoria than self-reported BIU.

### 3.2 Bayesian models

In the full and reduced Bayesian regression models for self-reported schistosomiasis and BIU (Table 1), odds were higher for lower degrees of urbanisation (i.e. urban < mixed rural/urban < rural wards). Increasing elevation and NDVI were negatively associated with both outcome variables in the reduced models. In the full models, the quadratic terms for elevation and NDVI were significant, indicating that the associations with the outcome variables were non-linear (though still negative over the range of observed values for both covariates). Amplitude of LST and NDVI, population density and LST were not significantly associated with the prevalence of self-reported schistosomiasis, but of these, the amplitude of LST was significantly and positively associated with self-reported BIU. In all models, variance of the SRE was greater than that of the NSRE, suggesting that there was a strong tendency towards clustering that was independent of the covariate effects. The DIC of the full models were marginally lower than for the reduced models (with a difference of <2 for both outcome variables), but as the difference was minimal we have opted to present results from the less complex reduced models hereafter.

### 3.3 Fitted values: point estimates

**Fig. 2:**
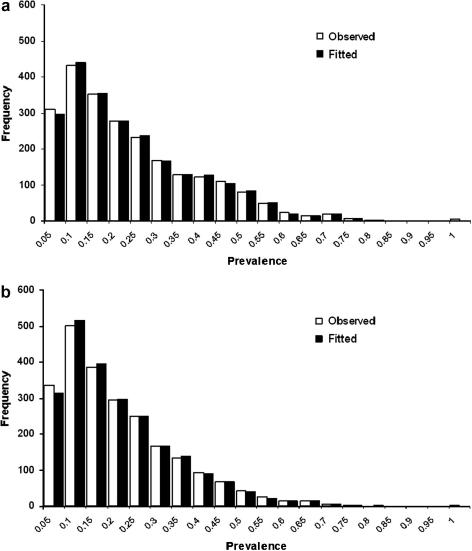
Frequency histograms of observed and fitted values using Bayesian spatial models for prevalence of self-reported schistosomiasis (a) and blood in urine (b) in Tanzanian wards.

Visual examination of maps (not shown) of the posterior median fitted prevalence values from the two reduced Bayesian models revealed that the spatial distributions were extremely similar to the raw prevalence values. This was indicative of the large sample sizes in most wards and the small amount of resultant smoothing that occurred using the Bayesian approach. However, examination of frequency histograms of observed and fitted values (Fig. 2) showed that some shrinkage was evident for the lower and upper tails of the ward-level prevalence distributions.

In relating these findings to the intervention strategy, none of the 558 wards that had observed prevalence of self-reported schistosomiasis >30% had fitted values that were below the threshold, but 11 of the 743 wards that had observed prevalence <10% had fitted values that were above the 10% threshold. For self-reported BIU, four of the 399 wards that had an observed prevalence >30% had fitted values that were below the threshold, and nine of the 839 wards that had an observed prevalence <10% intervention threshold had fitted values that were above the threshold. In total, 0.5% and 0.6% of wards would have a different status if the Bayesian smoothed point estimates were used instead of observed prevalence to define the intervention strategy according to self-reported schistosomiasis and BIU, respectively.

### 3.4 Fitted values: uncertainty measures

**Fig. 3:**
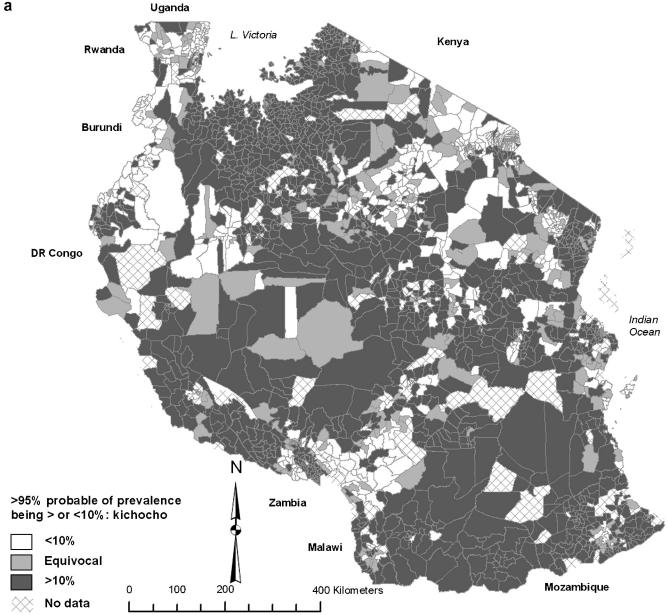

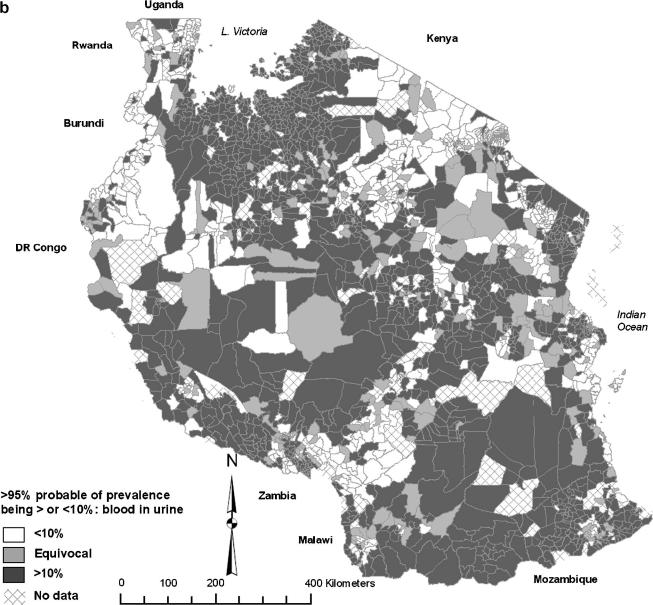
Bayesian probability maps of the prevalence of schistosomiasis (a) and blood in urine (b) using a posterior median prevalence threshold of 10%. Wards with >95% probability of having prevalence >10% are dark grey, wards with >95% probability of having prevalence <10% are white and wards with <95% probability of having prevalence > or <10% are light grey.

**Fig. 4:**
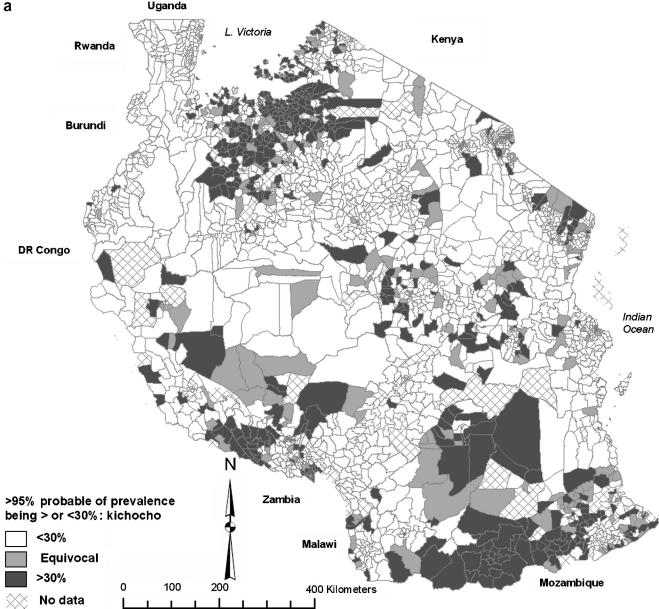

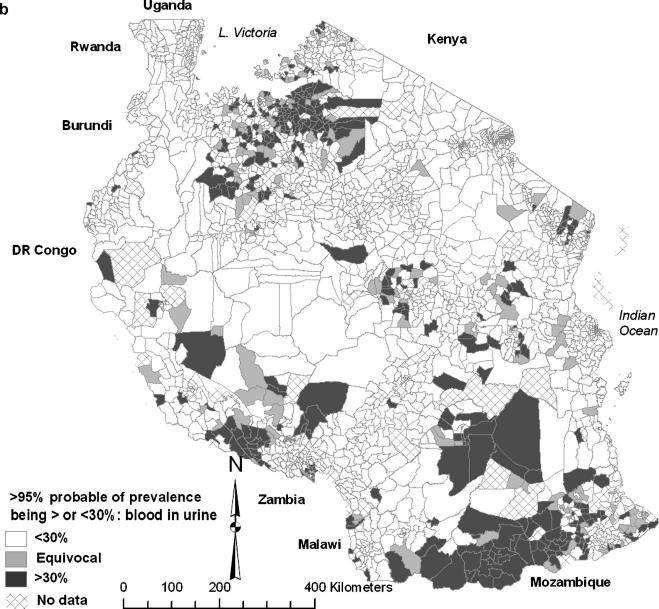
Bayesian probability maps of the prevalence of schistosomiasis (a) and blood in urine (b) using a posterior median prevalence threshold of 30%. Wards with >95% probability of having prevalence >30% are dark grey, wards with >95% probability of having prevalence <30% are white and wards with <95% probability of having prevalence > or <30% are light grey.

Bayesian probability maps are presented, which define those wards where the 95% Bayesian credible interval for the fitted prevalence values were wholly above or below the intervention thresholds of 10% (Fig. 3) and 30% (Fig. 4). Over half of all wards were >95% certain to have a prevalence of self-reported schistosomiasis and BIU that exceeded the 10% intervention threshold (Table 2). Most regions of mainland Tanzania contained wards with fitted prevalence estimates for self-reported schistosomiasis or BIU that were >95% certain of being >10%.

For the 30% threshold, 478 (20.5%) and 322 (13.8%) of wards were >95% certain of having a prevalence that exceeded 30% for self-reported schistosomiasis and BIU, respectively. Wards with >95% certainty of having fitted prevalence estimates >30% were grouped in north-western Tanzania (south and east of Lake Victoria) and border regions of south-western and south-eastern Tanzania, or were scattered through the central and north-eastern regions. Again, the eastern coast of Lake Victoria was the main area where differences occurred between self-reported schistosomiasis and BIU.

### 3.5 Random effects

**Fig. 5:**
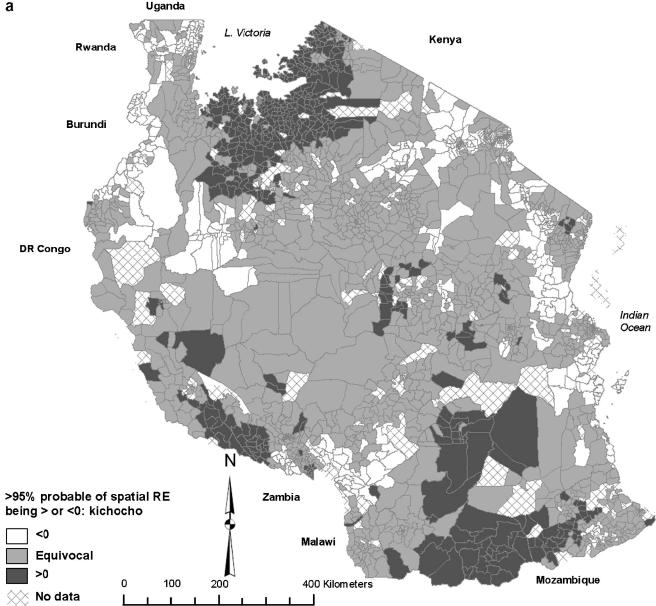

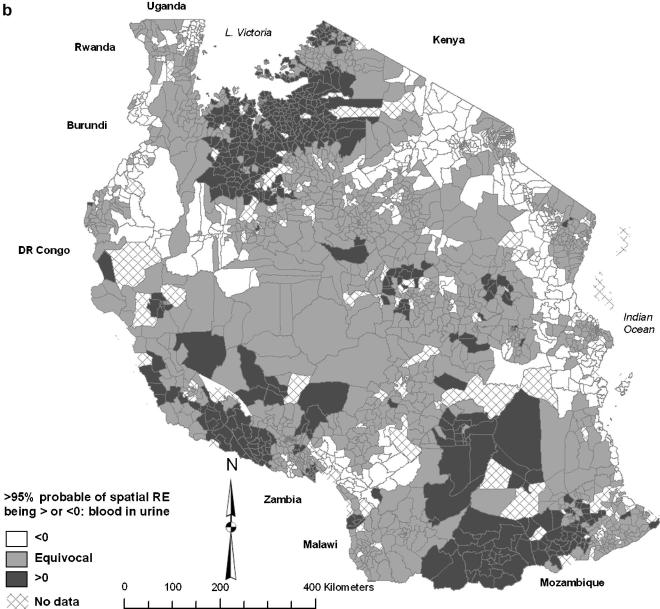
Probability maps of spatially structured residual components of Bayesian models for self-reported schistosomiasis (a) and blood in urine (b) in Tanzanian wards. Wards with >95% probability of having positive spatially structured residual prevalence (i.e. significant high-prevalence clusters) are dark grey, wards with >95% probability of having negative spatially structured residual prevalence (i.e. significant low-prevalence clusters) are white and wards with <95% probability of having positive or negative spatially structured residual prevalence are light grey.

Probability maps of the SRE (Fig. 5) highlighted wards >95% certain of having positive or negative values for spatially structured residual prevalence and therefore statistically significant clusters of high and low prevalence after accounting for the fixed effects. Visual examination of the map showed that significant high-prevalence clusters of self-reported schistosomiasis and BIU occurred in north-western, south-western, south-eastern, central and north-eastern Tanzania, and significant low-prevalence clusters occurred in western, northern and coastal regions and the southern highlands. As expected, maps of the NSRE showed a random pattern across the country (not shown).

## 4 Discussion

In the design of control programmes there is a requirement for evidence-based allocation of resources, including geographical targeting of the intervention. The current study represents an extensive spatial analysis of the largest ever schistosomiasis morbidity questionnaire survey conducted and is novel in that is was undertaken as part of the planning activities of a nation-wide schistosomiasis control programme. For a small number of wards (<1%), small sample sizes meant that prevalence estimates were statistically unstable and fitting a Bayesian model led to calculation of robust, smoothed estimates that altered the intervention status of the ward. More importantly, when considering the uncertainties surrounding the Bayesian smoothed point estimates, the intervention status of between 6.7% and 13.0% of wards was equivocal depending on the criterion used. This is relevant for two important reasons: firstly, it will help the NSSCP to prioritise its treatment programme towards wards that were certain to have a prevalence of schistosomiasis above the intervention thresholds and second, it might direct the programme coordinators to seek additional evidence to support the inclusion or exclusion of wards with equivocal intervention status prior to treatment.

The fact that almost all regions of Tanzania had wards that were certain to have a prevalence of schistosomiasis >10% means that virtually the whole country is in need of targeted treatment with praziquantel. Areas with a high certainty of having hyperendemic schistosomiasis (>30% according to self-reported schistosomiasis/BIU) were more localised and annual mass treatment of school-age children is only likely to be necessary in these foci, including parts of north-western Tanzania (already targeted by the SCI-supported programme), border areas of south-western and south-eastern Tanzania and isolated wards in central and north-eastern regions.

The significance of the fixed effects in the spatial models has been well documented (Sturrock, 1993). The degree of urbanisation is likely to be important due to its association with water contact and sanitation, with more urbanised areas being more likely to have a hygienic water supply. Rainfall (captured by NDVI in our models) has been demonstrated to influence the distribution of schistosomiasis via its influence on snail vector habitat suitability (Brooker and Michael, 2000). We hypothesise that the negative relationship between prevalence and NDVI may also arise due to more arid areas having increased schistosome contamination at more restricted, and therefore more heavily utilised, water contact sites. The negative relationship between prevalence and elevation may represent, in part, the association between high elevation and low temperatures, which are not suitable for snail survival, and also the possibility that water bodies at higher elevations provide less suitable snail habitats.

The SRE was interesting from an epidemiological viewpoint in that it highlighted clusters of high- and low-prevalence wards that were not explained by the fixed effects. Identification of these clusters may permit the generation of hypotheses regarding unmeasured but potentially important drivers of the spatial distribution of urinary schistosomiasis. A possible explanation for the clusters is that they relate to the distributions of the snail vector of *S. haematobium*. While ecological variables such as temperature and rainfall that were accounted for in the models are known to be important determinants of snail habitat suitability, other determinants (e.g. hydrological factors) are also likely to be important. Additional climatic, demographic and sociological factors might also influence the focal distribution of urinary schistosomiasis and more work needs to be done to define these associations.

Discriminatory performance of the questionnaire differed in different regions and this may have affected model estimates, particularly on the coast where discriminatory performance was below an acceptable level with respect to the 10% intervention threshold. Discriminatory performance of the questionnaire has been found in other studies to be lower in low-prevalence settings (Ansell et al., 1997) so these results were not unexpected. Also, a different comparator, prevalence of microhaematuria (itself a proxy measure for infection), was used on the coast, and this may have affected our calculations of discriminatory performance. A factor potentially affecting validity on the coast is ongoing treatment against schistosomiasis and other helminths, which requires further investigation. Discriminatory performance of the questionnaire with respect to the 50% threshold was high in north-western and coastal Tanzania. As future resources for schistosomiasis control in Tanzania are likely to be concentrated on hyperendemic regions, the questionnaire will be a useful tool for geographical targeting of resources. Validation has not yet been performed in many regions of Tanzania and this will be part of ongoing work of the NSSCP.

Another factor potentially affecting discriminatory performance was the non-specificity of the question about schistosomiasis (“kichocho”), which could have included cases of intestinal schistosomiasis caused by *S. mansoni*. We noted a different distribution of schistosomiasis compared with BIU along the coastline of Lake Victoria, which is an area characterised by high prevalence of *S. mansoni* infection (Clements et al., 2006a). The non-specificity of the term “kichocho” may also account, in part, for the overall higher prevalence of self-reported schistosomiasis than BIU. The schistosomiasis morbidity questionnaire is unproven for use in defining the prevalence of intestinal schistosomiasis and it was not an intention of our survey to define the distribution of this infection. However, as the basis of control is the same for intestinal and urinary schistosomiasis we did not consider this to be an important impediment for use of the questionnaire in planning the NSSCP.

Bayesian CAR models were appropriate as the questionnaire data were aggregated in small areas (wards), both necessarily, as the coordinates of all schools in Tanzania are not available, but also purposefully, as the ward was the administrative unit of the intervention. In a previous report, we used a Bayesian geostatistical model of parasitologically defined schistosome infection prevalence in north-western Tanzania, based on a point geo-referenced sample of schools, for the purposes of prediction in non-sampled locations (Clements et al., 2006a). Visual inspection of the maps derived from the questionnaire and geostatistical prediction in north-western Tanzania showed considerable similarity and these maps are being used as complimentary sources of evidence for planning the control programme in that area. It should be noted that different covariates were significant in the models, probably due to different outcome measures (one measured experiences of schistosomiasis and BIU, the other the presence of *S. haematobium* eggs in urine samples), spatial scales of analysis (one aggregated at the ward level, the other at the precise point location of the school being surveyed) and study areas (one investigated spatial relationships over several ecological zones, covering the entire country, the other investigated spatial relationships in a single zone in northwest Tanzania).

We attribute the success of the questionnaire survey to the fact that it was conducted via the highly organised Tanzanian education system and National School Health Programme which places a high priority on the need for control of schistosomiasis. While the generally large sample size in the vast majority of Tanzanian wards meant that raw estimates of prevalence derived from the national schistosomiasis questionnaire survey were robust, Bayesian modelling enabled the uncertainties surrounding point estimates to be fully appreciated. Identification of significant high- and low-prevalence clusters might lead to a greater understanding of the epidemiological processes involved in the commonly noted focal distribution of schistosomiasis. The maps derived from this analysis are being used by the NSSCP to identify wards where mass distribution of praziquantel is a priority. They have also been distributed to district officials, assisting with communication of the NSSCP plan at the district level. These maps will be available for ongoing planning and advocacy, with the aim of increasing long-term sustainability of national schistosomiasis control in Tanzania.

## Figures and Tables

**Table 1:** Bayesian spatial models of self-reported schistosomiasis and blood in urine in Tanzanian wards

Variable	Self-reported schistosomiasis						Self-reported BIU					
	Full model			Reduced model			Full model			Reduced model		
	OR mean	OR SD	OR 95% CI	OR mean	OR SD	OR 95% CI	OR mean	OR SD	OR 95% CI	OR mean	OR SD	OR 95% CI
Rural	1.445	0.170	1.146, 1.812	1.000			1.525	0.145	1.270, 1.846	1.000		
Mixed	1.292	0.146	1.045, 1.618	0.871	0.037	0.800, 0.944	1.351	0.121	1.127, 1.613	0.871	0.035	0.805, 0.944
Urban	1.000			0.601	0.051	0.512, 0.708	1.000			0.584	0.046	0.499, 0.681
Elevation	0.907	0.009	0.889, 0.924	0.926	0.007	0.912, 0.940	0.912	0.008	0.896, 0.928	0.926	0.007	0.913, 0.940
Elevation squared	1.004	0.001	1.003, 1.006				1.004	0.001	1.002, 1.005			
LST	1.015	0.016	0.984, 1.045				1.025	0.015	0.995, 1.056			
LST squared	1.001	0.002	0.998, 1.004				0.998	0.002	0.995, 1.001			
NDVI	0.965	0.044	0.878, 1.051	0.333	0.122	0.156, 0.623	0.968	0.041	0.891, 1.051	0.315	0.106	0.153, 0.570
NDVI squared	0.947	0.021	0.907, 0.989				0.977	0.020	0.938, 1.018			
Population density	0.96	0.018	0.927, 0.996				0.976	0.017	0.943, 1.011			
Population density squared	0.996	0.004	0.989, 1.003				0.998	0.003	0.991, 1.004			
Amplitude LST	1.045	0.024	0.999, 1.090				1.044	0.023	1.004, 1.092			
Amplitude NDVI	1.056	0.055	0.949, 1.169				1.068	0.053	0.967, 1.177			
												
	Beta mean	Beta SD	Beta 95% CI	Beta mean	Beta SD	Beta 95% CI	Beta mean	Beta SD	Beta 95% CI	Beta mean	Beta SD	Beta 95% CI
Intercept	−2.055	0.110	−2.270, −1.833	−1.610	0.016	−1.641, −1.577	−2.238	0.092	−2.429, −2.059	−1.763	0.015	−1.794, −1.734
Variance (NSRE)	0.301	0.024	0.256, 0.348	0.312	0.025	0.262, 0.359	0.246	0.020	0.206, 0.284	0.249	0.019	0.211, 0.289
Variance (SRE)	0.775	0.053	0.680, 0.889	0.741	0.034	0.674, 0.810	0.592	0.041	0.515, 0.678	0.599	0.028	0.543, 0.653
												
DIC	19,557.3			19,558.8			19,384.9			19,386.5		

OR, odds ratio; CI, credible interval; NDVI, normalised difference vegetation index; LST, land surface temperature; NSRE, non-spatial random effect; SRE, spatial random effect; DIC, deviance information criterion. Reference category for ward urbanisation category is urban in the full models and rural in the reduced models.

**Table 2:** Numbers of Tanzanian wards having posterior Bayesian estimates of prevalence of self-reported schistosomiasis and blood in urine that are certain to be above or below 10% and 30% intervention thresholds and numbers of wards with equivocal intervention status

Questionnaire response/intervention status	Numbers of wards (% of total)	
	10% intervention threshold	30% intervention threshold
*Self-reported schistosomiasis*		
>95% certain to be above threshold	1463 (62.7)	478 (20.5)
<95% certain to be above or below threshold^a^	302 (13.0)	192 (8.2)
>95% certain to be below threshold	567 (24.3)	1662 (71.3)
		
*Self-reported blood in urine*		
>95% certain to be above threshold	1348 (57.8)	322 (13.8)
<95% certain to be above or below threshold^a^	300 (12.9)	157 (6.7)
>95% certain to be below threshold	684 (29.3)	1853 (79.5)

a Equivocal intervention status.
