# Supplementary material for: Bayesian spatial analysis of a national urinary schistosomiasis questionnaire to assist geographic targeting of schistosomiasis control in Tanzania, East Africa
Source: Int J Parasitol. 2008 Mar;38(3-4):401–15. doi: 10.1016/j.ijpara.2007.08.001 (PMC2653941; doi:10.1016/j.ijpara.2007.08.001)
Supplement: Supplementary data [file mmc1.doc]

# DODOSO KUHUSU KICHOCHO KWA WANAFUNZI WA DARASA LA I, III NA V

Fomu hii ijazwe na Mwalimu wa Darasa, kwa kumhoji kila Mwanafunzi. Mwalimu aweke alama ya **√** kama jibu ni “ndiyo” au **0** kama “hapana” na **** kama “mwanafunzi hakumbuki au hawezi kujibu”. Lazima maswali yote yajibiwe. Kila safu ni kwa mwanafunzi mmoja tu. Kama visanduku vya ukurusa mmoja havitoshi kwa darasa, tumia ukurasa unaofuata. Fomu zilizojazwa zirudishwe kwa Mwalimu Mkuu, naye apeleke fomu za shule nzima kwa Mratibu wa Afya Shuleni, Ofisi ya Elimu - Wilaya. Asanteni. ('Tick' for yes, '0' for no and '-' for does not remember or cannot answer)

**Wilaya (District)_______ Kata (Ward)_________ Shule (School)__________ Darasa (Class)____ (I, III, au V)**

| Mwanafunzi (Student) | 1 | 2 | 3 | 4 | 5 | 6 | 7 | 8 | 9 | 10 | 11 | 12 | 13 | 14 | 15 | 16 | 17 | 18 | 19 | 20 | 21 | 22 | 23 | 24 | 25 | 26 | 27 | 28 | 29 | 30 | 31 | 32 | 33 | 34 | 35 |
| --- | --- | --- | --- | --- | --- | --- | --- | --- | --- | --- | --- | --- | --- | --- | --- | --- | --- | --- | --- | --- | --- | --- | --- | --- | --- | --- | --- | --- | --- | --- | --- | --- | --- | --- | --- |
| Umri (miaka) (Age) |  |  |  |  |  |  |  |  |  |  |  |  |  |  |  |  |  |  |  |  |  |  |  |  |  |  |  |  |  |  |  |  |  |  |  |
| Jinsia (MV au MS) (Sex) |  |  |  |  |  |  |  |  |  |  |  |  |  |  |  |  |  |  |  |  |  |  |  |  |  |  |  |  |  |  |  |  |  |  |  |
| **Swali la 1: Dalili ipi kati ya zifuatazo uliipata katika mwezi mmoja uliopita? Weka alama ya √ au 0 au  katika kisanduku kwa kila dalili** | | | | | | | | | | | | | | | | | | | | | | | | | | | | | | | | | | | |
| Kukohoa (Coughing) |  |  |  |  |  |  |  |  |  |  |  |  |  |  |  |  |  |  |  |  |  |  |  |  |  |  |  |  |  |  |  |  |  |  |  |
| Kuwashwa (Itching) |  |  |  |  |  |  |  |  |  |  |  |  |  |  |  |  |  |  |  |  |  |  |  |  |  |  |  |  |  |  |  |  |  |  |  |
| Maumivu ya kichwa (Headache) |  |  |  |  |  |  |  |  |  |  |  |  |  |  |  |  |  |  |  |  |  |  |  |  |  |  |  |  |  |  |  |  |  |  |  |
| Homa (Fever) |  |  |  |  |  |  |  |  |  |  |  |  |  |  |  |  |  |  |  |  |  |  |  |  |  |  |  |  |  |  |  |  |  |  |  |
| Maumivu ya tumbo (Stomach pain) |  |  |  |  |  |  |  |  |  |  |  |  |  |  |  |  |  |  |  |  |  |  |  |  |  |  |  |  |  |  |  |  |  |  |  |
| Damu katika mkojo (Blood in urine) |  |  |  |  |  |  |  |  |  |  |  |  |  |  |  |  |  |  |  |  |  |  |  |  |  |  |  |  |  |  |  |  |  |  |  |
| Damu katika kinyesi (Blood in stool) |  |  |  |  |  |  |  |  |  |  |  |  |  |  |  |  |  |  |  |  |  |  |  |  |  |  |  |  |  |  |  |  |  |  |  |
| Kuharisha (Diarrhoea) |  |  |  |  |  |  |  |  |  |  |  |  |  |  |  |  |  |  |  |  |  |  |  |  |  |  |  |  |  |  |  |  |  |  |  |
| **Swali la 2: Dalili ipi kati ya zifuatazo uliipata katika mwezi mmoja uliopita? Weka alama ya √ au 0 au  katika kisanduku kwa kila dalili** | | | | | | | | | | | | | | | | | | | | | | | | | | | | | | | | | | | |
| Malaria |  |  |  |  |  |  |  |  |  |  |  |  |  |  |  |  |  |  |  |  |  |  |  |  |  |  |  |  |  |  |  |  |  |  |  |
| Kuharisha (Diarrhoea) |  |  |  |  |  |  |  |  |  |  |  |  |  |  |  |  |  |  |  |  |  |  |  |  |  |  |  |  |  |  |  |  |  |  |  |
| Ugonjwa wa ngozi (Skin disease) |  |  |  |  |  |  |  |  |  |  |  |  |  |  |  |  |  |  |  |  |  |  |  |  |  |  |  |  |  |  |  |  |  |  |  |
| Ugonjwa wa macho (Eye disease) |  |  |  |  |  |  |  |  |  |  |  |  |  |  |  |  |  |  |  |  |  |  |  |  |  |  |  |  |  |  |  |  |  |  |  |
| Kichocho (Schistosomiasis) |  |  |  |  |  |  |  |  |  |  |  |  |  |  |  |  |  |  |  |  |  |  |  |  |  |  |  |  |  |  |  |  |  |  |  |
| Ugonjwa wa kifua (Chest infections) |  |  |  |  |  |  |  |  |  |  |  |  |  |  |  |  |  |  |  |  |  |  |  |  |  |  |  |  |  |  |  |  |  |  |  |
| Minyoo (Worms) |  |  |  |  |  |  |  |  |  |  |  |  |  |  |  |  |  |  |  |  |  |  |  |  |  |  |  |  |  |  |  |  |  |  |  |
| Matatizo ya tumbo (Stomach problems) |  |  |  |  |  |  |  |  |  |  |  |  |  |  |  |  |  |  |  |  |  |  |  |  |  |  |  |  |  |  |  |  |  |  |  |
